# Randomized, Double-Blind, Placebo-Controlled Trial to Test the Effects of a Nutraceutical Combination Monacolin K-Free on the Lipid and Inflammatory Profile of Subjects with Hypercholesterolemia

**DOI:** 10.3390/nu14142812

**Published:** 2022-07-08

**Authors:** Olga Protic, Raffaele Di Pillo, Alberto Montesanto, Roberta Galeazzi, Giulia Matacchione, Angelica Giuliani, Jacopo Sabbatinelli, Felicia Gurău, Andrea Silvestrini, Fabiola Olivieri, Roberto Antonicelli, Anna Rita Bonfigli

**Affiliations:** 1 Cardiology Unit, Italian National Research Center on Aging, IRCCS INRCA, 60127 Ancona, Italy; o.protic@inrca.it (O.P.); r.dipillo@inrca.it (R.D.P.); r.antonicelli@inrca.it (R.A.); 2 Department of Biology, Ecology and Earth Sciences, University of Calabria, 87036 Rende, Italy; alberto.montesanto@unical.it; 3 Clinical Laboratory and Molecular Diagnostic, Italian National Research Center on Aging, IRCCS INRCA, 60127 Ancona, Italy; r.galeazzi@inrca.it; 4 Department of Clinical and Molecular Sciences, DISCLIMO, Università Politecnica delle Marche, 60126 Ancona, Italy; g.matacchione@pm.univpm.it (G.M.); angelica.giuliani@staff.univpm.it (A.G.); j.sabbatinelli@pm.univpm.it (J.S.); gurau.felicia1@gmail.com (F.G.); a.silvestrini@pm.univpm.it (A.S.); f.olivieri@univpm.it (F.O.); 5 Center of Clinical Pathology and Innovative Therapy, Italian National Research Center on Aging, IRCCS INRCA, 60127 Ancona, Italy; 6 Scientific Direction, Italian National Research Center on Aging, IRCCS INRCA, 60127 Ancona, Italy

**Keywords:** dietary supplement, nutraceutical combination, monacolin K-free, lipid profile, inflammatory profile, hypercholesterolemia

## Abstract

Background: Nutraceutical combinations (NCs) against hypercholesterolemia are increasing in the marketplace. However, the availability of NCs without monacolin K is scarce even though the statin-intolerant population needs it. Methods: This study is a parallel-group, randomized, placebo-controlled, double-blind trial. We evaluated the effects of the NC containing phytosterols, bergamot, olive fruits, and vitamin K_2_ on lipid profile and inflammatory biomarkers in 118 subjects (mean age ± SD, 57.9 ± 8.8 years; 49 men and 69 women) with hypercholesterolemia (mean total cholesterol ± SD, 227.4 ± 20.8 mg/dL) without clinical history of cardiovascular diseases. At baseline and 6 and 12 weeks of treatment, we evaluated lipid profile (total, LDL and HDL cholesterol, and triglycerides), safety (liver, kidney, and muscle parameters), and inflammatory biomarkers such as hs-CRP, leukocytes, interleukin-32, and interleukin-38 and inflammatory-microRNAs (miRs) miR-21, miR-126, and miR-146a. Results: Compared to the placebo, at 6 and 12 weeks, NC did not significantly reduce total cholesterol (*p* = 0.083), LDL cholesterol (*p* = 0.150), and triglycerides (*p* = 0.822). No changes were found in hs-CRP (*p* = 0.179), interleukin-32 (*p* = 0.587), interleukin-38 (*p* = 0.930), miR-21 (*p* = 0.275), miR-126 (*p* = 0.718), miR-146a (*p* = 0.206), myoglobin (*p* = 0.164), and creatine kinase (*p* = 0.376). Among the two reported, only one adverse event was probably related to the nutraceutical treatment. Conclusions: The evaluated nutraceutical combination did not change serum lipid profile and inflammatory parameters, at least not with the daily dose applied in the present study.

## 1. Introduction

Cardiovascular diseases (CVDs) are the leading cause of death worldwide [1]. Hypercholesterolemia is the major cardiovascular risk factor associated with an inflammatory process that contributes to atherosclerotic plaque formation [2]. According to NCEP ATPIII guidelines, borderline high levels of total cholesterol range from 200 to 240 mg/dL, and high levels are considered ≥240 mg/dL [3]. The management of serum cholesterol levels is a central objective in preventing cardiovascular events. Lifestyle changes and pharmacological intervention are the strategies against elevated plasma low-density lipoprotein (LDL) cholesterol, the major contributor to atherosclerotic cardiovascular disease [4]. However, many people refuse pharmacological treatment for their possible side effects and personal reasons. Drug therapy in moderately hypercholesterolemic subjects in primary prevention is still debated. This issue derives from the scarce evidence of statin’s beneficial effects on a healthy population in primary cardiovascular disease prevention, but the risk of their adverse effects is possible [5].

There is a need for an alternative approach. The European Society of Cardiology guidelines indicate lipid-lowering functional foods or nutraceuticals for managing dyslipidemia [6]. Randomized Clinical Trials (RCTs) have already demonstrated the efficacy of single nutraceuticals such as red yeast rice (RYR), phytosterols, berberine, soy, fibers, and other lipid-lowering nutraceuticals [7,8].

The advantage of nutraceutical combinations versus single nutraceuticals is their possible additive or synergistic effects. Moreover, they can affect different targets involved in the atherosclerotic process, such as lipid metabolism, oxidative stress, and inflammation.

A rising tide of nutraceutical combinations against hypercholesterolemia is arriving on the market. Interestingly, 80% of these NCs contain RYR component monacolin K “natural statin” in their formulations. The adverse effects of the monacolin K are similar to those of synthetic statins [9]. EFSA Panel on Food Additives and Nutrient Sources added to Food reported that no dietary intake of monacolin from RYR has been found that does not raise concerns about its harmful effects [10].

It seems there is an underestimation of adverse events from RYR, because nutraceutical compounds are natural products but not synthetic ones. They usually are considered “safe” and without adverse effects. Considering all above mentioned comments, RYR-intolerant populations need to have the availability of monacolin K-free NCs in the marketplace.

For these reasons, this work aims to investigate the cholesterol-lowering effects of one NC monacolin K-free, containing phytosterols, bergamot, olive fruit, and vitamin K2, on subjects with hypercholesterolemia.

## 2. Materials and Methods

### 2.1. Study Design

This study was designed as a randomized, double-blind, placebo-controlled, parallel-group clinical trial. The study was conducted in accordance with the Declaration of Helsinki. The Ethical Committee of the IRCCS INRCA, Ancona, Italy, approved the protocol, and all the participants provided written informed consent before inclusion in the study. The study has been registered in the Australian New Zealand Clinical Trials Registry (reference no.: ACTRN12619000170123). The study protocol was previously described in detail [11]. Here, we report the summary of the study methods.

Participants were randomized by the physician into two parallel treatment arms. A blinded treatment kit containing test or placebo capsules was distributed. The NC and placebo capsules were randomly assigned to the test and placebo groups at a 1:1 ratio according to the random number that the statistician created throughout SPSS ver. 25.0 software package (IBM Corp., Armonk, NY, USA).

### 2.2. Participants

The clinical trial was conducted on the planned 125 subjects with hypercholesterolemia, with total serum cholesterol levels ≥200 and ≤250 mg/dL.

The participants included men and women subjects of 40 years or over in primary prevention for cardiovascular disease. The exclusion criteria were as follows: treatment with statins or lipid-lowering food supplements, hypothyroidism or hyperthyroidism, diabetes, intestinal malabsorption, acute illnesses, neoplastic diseases or less than one-year life expectancy, statin non-related liver disease, severe chronic renal failure, presumed pregnancy and pregnancy planning, therapy with anticoagulants, presence of cognitive disorders and other impediments that do not guarantee the correct adherence to the study treatments, allergy/intolerance to one or more components of dietary supplement or placebo, participation in another clinical trial of intervention in the previous 3 months, and incapacity, impossibility, or unavailability to sign the written consent.

The participants were recruited to the outpatient facilities of the Cardiology Unit of IRCCS INRCA (Ancona, Italy) between 6 March 2018 and 16 January 2020. The date of the last follow-up data collection was 20 April 2020. At the screening visit, subjects who met the inclusion criteria signed a written informed consent offered by a physician. At the baseline visit, the participants were randomized according to the 1:1 ratio in the intervention group/BruMeChol™ or placebo group. Both NC group and placebo were treated with two indistinguishable capsules of NC or placebo/day, one capsule 15 min before lunch and one before dinner. The treatment was performed for three months. The study scheduled a follow-up after 6 and 12 weeks.

After the participant signed informed consent, demographic data were collected at the screening visit. Biomedical parameters of total cholesterol, fasting glucose, liver profile including aspartate aminotransferase (AST) and alanine aminotransferase (ALT), kidney profile including creatinine and calculation of estimated glomerular filtration rate by CKD-EPI equation (CKD-EPI e-GFR), and thyrotropin (TSH) were measured.

### 2.3. Interventions

NC consists of 400 mg phytosterols, 100 mg bergamot fruit extract (*Citrus bergamia*), 20 mg olive fruit extract *(Olea europea)*, and 52 µ vitamin K2. The placebo capsule consists of 2% of magnesium stearate, 2% of silicon dioxide, and 96% of calcium phosphate. The placebo was manufactured to have a similar appearance, shape, weight, taste, and color as NC’s capsule.

Baseline, 6 weeks, and 12 weeks from randomization included biochemical measurements such as the following: serum lipid profile (total, LDL, HDL cholesterol, and triglycerides,) liver profile including (AST and ALT) kidney profile (creatinine and CKD-EPI e-GFR), fasting glucose, fool blood exam (FBE), leukocyte formula, electrolytes, cardiac/muscular markers creatine kinase (CK) and myoglobin, *hs*-C-reactive protein (CRP), and Lipoprotein(a) (Lp(a)). All these measurements were determined by standard laboratory methods.

Furthermore, interleukins (IL-32 and IL-38) and inflammatory-miRs (miR-21, miR-126, and miR-146a) were examined. For miRs determination, total RNA was isolated from samples (100 μL) using Total RNA Purification Kit (product #17200) by Norgen Biotek Corporation (Thorold, ON, Canada), according to the manufacturer’s specific recommendations. RNA was stored at −80 °C until use. Selected miRNAs were reverse transcribed using the TaqMan miRNA Reverse Transcription Kit (Life Technologies, Carlsbad, CA, USA) as recommended by the manufacturer and quantified by a miRNA assay (Applied Biosystems, Foster City, CA, USA). Relative quantification was performed using the 2^−ΔCq^ method (ΔCq = CqmicroRNA-Cqcel-miR-39-3p). Synthetic cel-miR-39-3p was spiked-in before RNA isolation for normalization in subsequent qRT-PCR.

Human IL1F10 (IL-38) ELISA Kit was purchased from Wuhan Fine Biotech Co. Plasma IL-38 concentration was determined in triplicate according to the manufacturers’ instructions. The mean intra-assay coefficient of variation (CV) for IL-38 measurements was 2.4% ± 1.3%.

Human IL-32 ELISA Kit was purchased from Antibodies-online.com (accessed on 15 June 2019), Aachen, Germany. Plasma IL-32 concentration was determined in triplicate according to the manufacturers’ instructions. The mean intra-assay CV for IL-32 measurements was <10%.

Anthropometric measurements such as weight, height, waist, and hip circumference were performed and repeated on each successive visit for the participants.

Medical history data were taken on the baseline visit and 12 weeks’ completion visit: hypertension, diabetes, hypertriglyceridemia, hyperlipidemia, and familiarity for cardiovascular diseases, anemia, chronic renal failure, chronic respiratory failure, mental illness, digestive system diseases, liver disease, drug allergies, and surgical interventions. In addition, at baseline and 12 weeks, visit pressure and electrocardiography were measured. The physical activity was followed by using the International Physical Activity Questionnaire (IPAQ-SF) short form [12].

### 2.4. Statistical Analyses

For this study design, statistical calculations indicated that a total of 125 participants must be included in this study design with a dropout rate of 5%. Thus, 118 subjects must be recruited (at least 59 for the treatment arm) with a power of 0.85 and a type I error probability of 0.05.

Demographic, clinical characteristics, and outcomes data were summarized with counts and percentages for categorical variables, means (standard deviations) for normally distributed continuous variables, and medians (with interquartile ranges) for other continuous variables. The Student’s *t*-test and the corresponding nonparametric Wilcox tests (as appropriate) were used to compare the values of quantitative variables between placebo and treatment groups. The Chi-square test was used to compare the values of categorical variables. The compliance with the treatment was assessed, and this variable was included in the analysis. Repeated measures analysis of variance (ANOVA) was used to test whether treatment (between-subjects factor) differentially affects the mean values of cholesterol levels (within-subjects factor) over time. The same analysis was repeated for the other lipid profile parameters (LDL and HDL cholesterol and triglycerides) as well as safety parameters (e.g., AST/GOT and ALT/GPT), kidney profile (creatinine and CKD-EPI e-GFR) and inflammatory biomarkers (e.g., CRP, Lp(a), IL-32, IL-38, miR-21, miR-126, and miR-146a) that were used as within-subjects’ factors. Mauchly’s test was used to assess the assumption of sphericity, and Greenhouse–Geisser correction was adopted for violations of this assumption. ANOVA tests were followed by pairwise comparisons among experimental times carried out using the Wilcox method. A significant level of 0.05 was used throughout the study. All analyses were performed with SPSS statistical software ver. 25.0 (IBM Corp., Armonk, NY, USA).

## 3. Results

**Figure 1:**
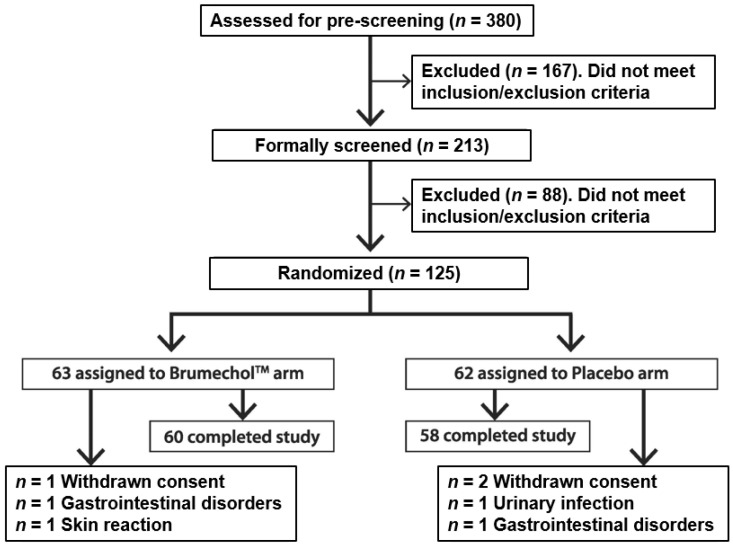
Flow chart of participants.

A total of 380 candidates were pre-screened for eligibility. Among these candidates, 213 were formally screened after they signed informed consent. After inclusion/exclusion criteria verification, 125 subjects were enrolled in the study. The participants were randomized into BruMeChol^TM^ (*n* = 63) and placebo (*n* = 62) arms (Figure 1). The details of the study flow diagram are described in Figure 1.

Three participants in the BruMeChol^TM^ and four in the placebo arm withdrew before study completion (Figure 1). In particular, three subjects did not conclude the study for personal reasons and four for adverse events (AEs). Two subjects were withdrawn for gastrointestinal disorders, one for urinary infection and one for skin reaction. Of the four AEs, three AEs were not related to the study, while one AE in skin reaction was considered possibly related to study treatment.

Ninety-nine subjects (79.2%), forty-eight in the active treatment group and fifty-one in the placebo group, were classified as compliant (compliance levels between 80% and 120%).

The baseline characteristics of the participants who completed the study (*n* = 118) are described in Table 1.

There is no significant difference between the placebo and active treatment groups in demographic, anthropometric, and inflammatory profiles, showing that the two groups were well balanced. Regarding lipid profile, a significant difference has been found only for the total/HDL cholesterol ratio (Table 1). Nevertheless, despite this difference, both groups’ total/HDL cholesterol ratio remains in the range of values that indicate low cardiovascular risk [13].

Table 2 shows the baseline values and 6- and 12-week’s levels of safety laboratory analyses, including liver, renal, and muscle parameters in participants with compliance higher than 80%. No statistically significant differences in these parameters were observed during the study.

No significant reduction was observed in total cholesterol, HDL-c, LDL-c, and triglycerides levels in the nutraceutical group at 6 and 12 weeks compared to the placebo group (Table 3). No change in physical activity evaluated by the IPAQ test was found (Table 3). No significant pairwise differences were also detected for each experimental time point using the Wilcox test (*p* > 0.05 for all pairwise comparisons). 

No statistically significant differences were observed concerning the inflammatory parameters after 12 weeks in the nutraceutical group compared to the placebo group (*p* > 0.05 for all; Table 4).

## 4. Discussion

There is scarce clinical evidence of NC effects performed by RCT in hypercholesterolemic populations [14]. Most of these NCs contain monacolin K, a “natural statin’’ component of RYR in their combinations [14]. Many patients taking statins report muscle-related symptoms, one of the most important causes of statin treatment discontinuation, associated with an increased risk of cardiovascular events [15]. Thus, it is relevant for statin-intolerant populations to find efficient NCs without monacolin K. For these reasons, we chose to study the safety and efficacy of an NC that does not contain monacolin K containing phytosterols, bergamot, olive fruits, and vitamin K_2_. This is the first RCT that investigates the safety and effects of BruMeChol^TM^ in hypercholesterolemic subjects.

The mechanism of action of phytosterols lies in the base of their structural similarity with cholesterol. When ingested with plant foods, cholesterol absorption is reduced [16]. Besides their lipid-lowering effects, plant sterols have anti-inflammatory properties [17]. It is well described that nutraceutical bergamot *Citrus Bergamia*, besides its lipid-lowering effect, also has anti-oxidative and anti-inflammatory properties [18,19,20,21]. The beneficial effects of *Olea Europea* derive from its components hydroxytyrosol and tyrosol phenolic antioxidants [22]. This molecule can prevent LDL oxidation, a critical step in the initial process of atherosclerosis [23]. The role of vitamin K in chronic diseases such as inflammation, cardiovascular disease, and osteoarthritis has been described [24]. Vitamin K has anti-inflammatory and anti-oxidative effects by blocking reactive oxygen species generation [25].

Due to each compound’s antioxidant and anti-inflammatory properties, it is possible to hypothesize that, besides its possible lipid-lowering effects, this combination could positively affect the inflammatory process, a key event in atherosclerotic plaque formation. Changes in the levels of circulating inflammatory marker hs-CRP [26] and inflammatory cytokines such as interleukin-32 (IL-32) [27,28,29] and interleukin-38 (IL-38) [30,31], as well as a variation in the expression of specific circulating inflamma-miRs (miR-21, miR-146a and miR-126) [32], have been well described in CVDs. For these reasons, we chose to also investigate BruMeChol^TM^ effects on these parameters.

Although a single compound contained in this NC exhibits beneficial effects on lipid traits and inflammatory status, the results of the RCT showed that 12-week treatment with the tested NC was not able to exert lipid-lowering effects in hypercholesterolemic subjects. Several issues might explain these unexpected results: doses of single compounds, possible antagonistic interactions between the single compounds rather than the expected additive or synergistic effects of the single compounds, and/or the non-optimal bioavailability of the NC in vivo.

Several data suggest that these compounds exert a pro-healthy effect modulating lipid traits. The NCEP/ATP III recommendation affirmed that the administration of phytosterols <2 g/day is less effective in reducing LDL levels than higher doses [33]. Indeed, most guidelines on dyslipidemia treatment and prevention reported that the intake of almost 2 g/day of phytosterols reduces 10% LDL-cholesterol associated with lifestyle changes [34,35,36]. BruMeChol^TM^ contains 400 mg of phytosterols taken twice/day, resulting in less than the recommended doses.

It was demonstrated that nutraceutical bergamot (*Citrus Bergamia*) in doses ranging 500–1500 mg/day was associated with an LDL-C reduction from 7.6 to 40.8% [18]. Moreover, Toth et al. demonstrated that the daily use of 150 mg of flavonoids from bergamot reduces total cholesterol (−13.2%) and LDL cholesterol (−18.2%) [37]. Each capsule of BruMeChol^TM^ contains 100 mg of bergamot fruit (98 mg of flavonoids) taken twice/day. However, even though this dose of flavonoids was higher than reported by Toth et al. NC BruMeChol did not show a lipid-lowering effect. The effect of a single compound in lipid-lowering could be different when it is combined with other compounds.

Regarding olive fruit, epidemiological evidence from observational studies suggests that dietary polyphenols (PPs) can help manage the main risk factors for cardiovascular and metabolic diseases, such as hyperglycemia and dyslipidemia [38]. Lockyer et al. reported that the use of phenolic-rich olive leaf extract containing 136 mg of oleuropein and 6 mg of hydroxytyrosol reduces total plasma cholesterol (−0.32 mmol/L, *p* = 0.002) and LDL cholesterol (−0.19 mmol/L, *p* = 0.017) [39]. BruMeChol^TM^ contains 20 mg of *Olea Europea* (19.6 mg polyphenols, from which 6 mg of hydroxytyrosol) taken twice/day.

Regarding vitamin K_2_, literature data reported adequate intakes ranging from 55 to 90 mg/day for women and from 65 to 120 mg/day for adult men [40]. BruMeChol^TM^ capsule contains 52 µ of vitamin K_2_, twice/day.

Considering all above mentioned observations, one reason for the lack of a desirable lipid-lowering effect of BruMeChol^TM^ could be that the chosen concentration of single nutraceuticals does not achieve the threshold of effectiveness.

Otherwise, the potential interaction among the nutraceuticals in the mixture could affect their activity. In fact, different types of compounds could exert additive, synergistic, or antagonistic effects when combined [41]. Evaluating the interaction among single natural compounds in vivo is not easily achievable. In vitro, effortful cell-based assays must be performed to acquire valuable data on combination effects in NC [41]. In the BruMeChol^TM^ case, it seems that there is a lack of desired additive or synergistic effects. Only a specifically tailored study could answer to this question.

The bioavailability of NCs is another important parameter that needs to be taken into consideration. It is known that the bioavailability of phytosterols and polyphenols is a critical point that could limit the lipid-lowering effects as well as anti-inflammatory activity [42,43].

Moreover, the limitation of this study is that there is no evidence of the participant’s nutrient intake due to the lack of a nutritional questionnaire. However, the results showed no changes in weight, waist, and physical activity measured by IPAQ test. In addition, subjects were previously advised not to change their lifestyle habits.

Since many studies reported the capacity of nutraceuticals described above to modulate inflammatory parameters, we analyzed specific circulating cytokines and microRNAs associated with the inflammatory process [44,45]. No significant modulation of some inflammatory parameters was observed in subjects treated for 12 weeks with the investigated NC compared to the placebo group. It is probable that the lack of anti-inflammatory effects derives from the same causes discussed above.

Data reported in Table 1 clearly show that the two groups were well balanced for all the analyzed parameters and characteristics, with the only exception of the total/HDL cholesterol ratio, which was confirmed by the association results reported in Table 3. Since the primary aim of the study was to investigate the efficacy and safety of BruMeChol™ NC to reduce total cholesterol levels in serum of patients with hypercholesterolemia, no randomization procedure could avoid the unbalance relieved for this parameter. A slightly different consideration could be carried out for CDK-EPI eGFR at baseline. Using eGFR in its quantitative scale, a statistically significant difference between the analyzed groups was detected (Table 2, *p* Groups = 0.038). However, more than 90% of participants in both placebo (90.2%) and BruMeChol™ (91.7%) groups were stratified in the same eGFR stage (stage 2: 60–90 mL/min/1.73 mq), which indicates that they belong to the same risk category. Thus, this baseline difference can be considered negligible.

In conclusion, even though the literature evidence for a single compound present in the studied NC demonstrated beneficial effects on lipid and inflammatory profile, the results of this RCT showed that the studied NC did not change these parameters.

There is a need for alternative, effective, and safe NCs for treating patients affected by hypercholesterolemia, especially for the subject’s intolerant to monacolin K. This review offers a picture of the main scientific RCTs evidence to evaluate NCs as an alternative approach also in subjects that refuse lipid-lowering drug therapy.

It is necessary to provide in-depth and adequate studies to develop efficient and safe NCs from preclinical to clinical research including RCTs as the final step. The nutraceutical candidates should be selected based on their molecular mechanism of action, biological activity, types of interaction (such as additive, synergistic, or antagonistic), and clinical data. Studies addressing all these issues should be encouraged.

New nutraceuticals can play an important role in maintaining the health status of humans, especially in certain categories of patients (for example, statin-intolerant), but this could be verified only if novel NC will be previously studied by RCTs to demonstrate its safety and effectiveness. This research is important not only for the monacolin K intolerant population but also could be useful for the subjects with hypercholesterolemia that refuse lipid-lowering drug therapy.

## Figures and Tables

**Table 1:** Baseline clinical and biochemical characteristics of the enrolled subjects who completed the study by treatment group.

	NC *n* = 59	Placebo *n* = 59	*p*
Age (years)	56.59 (9.55)	59.27 (7.91)	0.100
Gender (men)	24 (40.7)	25 (42.4)	0.852
Weight (kg)	73.11 (15.37)	70.99 (13.39)	0.426
Height (cm)	168 (160–175)	166 (160–173)	0.599 *
Waist circumference (cm)	92.65 (10.66)	91.90 (10.65)	0.704
Hip circumference (cm)	103.23 (8.68)	103.09 (8.64)	0.932
Body Mass Index (kg/m^2^)	25.4 (23.0–29.7)	24.5 (22.2–28.6)	0.236 *
Systolic Blood Pressure (mm/Hg)	130 (115–140)	130 (120–149)	0.220 *
Diastolic Blood Pressure (mm/Hg)	80 (72–85)	80 (70–85)	0.605 *
Heart rate (bpm) *	66 (60–70)	67 (61–72)	0.287 *
Fasting Glucose (mg/dL)	95.08 (8.62)	96.17 (10.66)	0.544
Total Cholesterol (mg/dL)	225.69 (19.55)	229.02 (22.04)	0.388
Cholesterol HDL (mg/dL)	62.03 (16.86)	68.20 (15.98)	0.044
Cholesterol LDL (mg/dL)	156.29 (21.61)	156.53 (19.53)	0.950
Total / HDL Cholesterol (mg/dL)	3.74 (3.06–4.57)	3.40 (3.01–3.96)	0.079 *
Triglycerides (mg/dL)	105 (73–148)	95 (70–143)	0.557 *
AST (U/L)	19 (16–21)	18 (16–22)	0.627 *
ALT (U/L)	15 (13–23)	16 (13–24)	0.760 *
Creatinine (mg/dL)	0.8 (0.7–1.0)	0.9 (0.8–1.0)	0.779 *
CKD-EPI eGFR (mL/min/1.73 mq)	86 (80–90)	84 (74–90)	0.167 *
*hs*-C-reactive protein (mg/dL)	0.09 (0.05–0.20)	0.12 (0.06–0.27)	0.387 *
Lipoprotein (a) (mg/dL)	104 (48–299)	200 (49–509)	0.228 *
Creatine kinase (U/L)	98 (70–134)	102 (78–134)	0.794 *
Leukocytes (×10^3^/L)	5.89 (5.01–6.75)	5.87 (5.06–6.56)	0.655 *

Mean (standard deviation (SD)) and median (interquartile range (IQR)) for description of normally and non-normally distributed data, respectively; categorical variables are described as *n* (%). NC: nutraceutical combination BruMeChol^TM^; LDL: low-density lipoprotein; HDL: high-density lipoprotein; AST: aspartate aminotransferase; ALT: alanine aminotransferase; CKD-EPI eGFR: calculation of estimated glomerular filtration rate by CKD-EPI equation. *p*-value for comparisons between groups by Chi-square test for categorical variables and t-test or Wilcox test (as appropriate) for continuous variables. * Wilcox test *p*-value.

**Table 2:** Baseline and 6- and 12-week’s levels in safety parameters in participants who completed the study by group allocation.

	Baseline	6th Week	12th Week	*p* Group	*p* Time	*p* Interaction
**AST** (U/L)						
NC	19.5 (4.7)	20.2 (5.8)	20.2 (6.1)	0.986	0.112	0.906
Placebo	19.5 (5.5)	20.0 (5.5)	20.3 (6.4)			
**ALT** (U/L)						
NC	18.4 (8.6)	21.0 (13.2)	19.5 (10.2)	0.975	0.218	0.207
Placebo	19.5 (10.8)	19.5 (11.2)	19.7 (10.9)			
**Creatinine** (mg/dL)						
NC	0.87 (0.15)	0.87 (0.16)	0.86 (0.14)	0.240	0.200	0.581
Placebo	0.90 (0.22)	0.92 (0.20)	0.90 (0.21)			
**CDK-EPI eGFR** (ml/min/1,73 mq)						
NC	82.6 (9.75)	81.2 (12.4)	83.3 (7.7)	0.057	0.094	0.784
Placebo	79.7 (12.7)	77.7 (12.1)	79.1 (12.5)			
**Creatine kinase** (U/L)						
NC	120.1 (95.6)	122.8 (113.3)	125.9 (138.0)	0.798	0.079	0.376
Placebo	111.3 (47.8)	114.4 (50.2)	130.6 (86.1)			
**Myoglobin** (ng/mL)						
NC	35.2 (25.6)	35.0 (32.3)	33.9 (25.1)	0.998	0.412	0.164
Placebo	35.5 (17.9)	33.0 (16.4)	35.6 (19.0)			

Values are means (SD). NC: nutraceutical combination BruMeChol^TM^; AST: aspartate aminotransferase; ALT: alanine aminotransferase; CKD-EPI eGFR: calculation of estimated glomerular filtration rate by CKD-EPI equation; *p*-value for one-way repeated measures ANOVA for continuous variables (group = treatment allocation; time = baseline, 6 weeks, and 12 weeks), *p* < 0.05, by ANOVA simple effect analyses with Bonferroni correction.

**Table 3:** Baseline and 6- and 12-week’s levels in lipid profile and IPAQ values by group allocation.

	Baseline	6th Week	12th Week	*p* Group	*p* Time	*p* Interaction
**Total cholesterol** (mg/dL)						
NC	225.9 (19.7)	224.9 (21.6)	229.9 (25.5)	0.855	0.476	0.083
Placebo	228.6 (22.0)	225.6 (24.9)	224.5 (24.7)			
**Cholesterol LDL** (mg/dL)						
NC	156.9 (21.3)	155.8 (22.6)	158.8 (25.4)	0.384	0.410	0.150
Placebo	156.4 (19.7)	153.2 (22.7)	152.1 (23.1)			
**Cholesterol HDL** (mg/dL)						
NC	61.7 (16.8)	62.2 (16.4)	62.7 (17.6)	0.078	0.880	0.158
Placebo	68.1 (16.1)	67.6 (14.8)	66.8 (14.4)			
**Total/HDL Cholesterol** (mg/dL)						
NC	3.9 (1.1)	3.8 (1.0)	3.9 (1.2)	0.024	0.144	0.379
Placebo	3.5 (0.8)	3.5 (0.8)	3.5 (0.8)			
**Triglycerides** (mg/dL)						
NC	115.6 (57.5)	120.1 (68.2)	132.1 (81.8)	0.364	0.032	0.822
Placebo	107.8 (49.2)	110.6 (77.3)	118.0 (78.8)			
**IPAQ**						
NC	1384.1 (1911.4)	-	1306.4 (1683.9)	0.379	0.425	0.841
Placebo	1127.1 (1242.8)	-	1080.6 (1154.9)			

Values are means (SD). NC: nutraceutical combination BruMeChol^TM^; LDL: low-density lipoprotein; HDL: high-density lipoprotein; IPAQ: International Physical Activity Questionnaire. *p*-value for one-way repeated measures ANOVA for continuous variables (group = treatment allocation; time = baseline, 6 weeks, and 12 weeks), *p* < 0.05, by ANOVA simple effect analyses with Bonferroni correction.

**Table 4:** Inflammatory parameters by group allocation.

	Baseline	6th Week	12th Week	*p* Group	*p* Time	*p* Interaction
**Leukocytes (×10^3^/µL)**						
NC	6.1 (1.8)	6.4 (1.4)	6.1 (1.5)	0.289	0.215	0.207
Placebo	5.8 (1.2)	5.9 (1.3)	6.1 (1.6)			
**Neutrophils (×10^3^/µL)**						
NC	3.3 (1.3)	3.4 (1.0)	3.3 (1.1)	0.764	0.243	0.105
Placebo	3.2 (1.0)	3.2 (1.0)	3.5 (1.3)			
C-reactive protein (mg/dL) *						
NC	−0.98 (0.45)	−0.93 (0.52)	−0.96 (0.43)	0.694	0.488	0.179
Placebo	−0.92 (0.50)	−0.98 (0.44)	−0.88 (0.48)			
**Interleukin-32 (pg/mL)**						
NC	39.97 (56.60)	-	44.26 (57.23)	0.408	0.452	0.587
Placebo	58.61 (82.28)	-	59.31 (96.92)			
**Interleukin-38 (pg/mL)**						
NC	186.98 (124.11)	-	183.38 (122.10)	0.095	0.667	0.930
Placebo	249.08 (147.76)	-	246.70 (134.76)			
**miR-21**						
NC	4.53 × 10^−7^ (2.44 × 10^−7^)	-	4.47 × 10^−7^ (3.31 × 10^−7^)	0.923	0.427	0.275
Placebo	4.34 × 10^−7^ (2.39 × 10^−7^)	-	4.77 × 10^−7^ (3.01 × 10^−7^)			
**miR-126**						
NC	1.82 × 10^−7^ (1.51 × 10^−7^)	-	1.76 × 10^−7^ (1.60 × 10^−7^)	0.578	0.280	0.718
Placebo	1.68 × 10^−7^ (1.55 × 10^−7^)	-	1.55 × 10^−7^ (1.07 × 10^−7^)			
**miR-146a**						
NC	2.23 × 10^−8^ (9.02 × 10^−9^)	-	2.11 × 10^−8^ (1.04 × 10^−8^)	0.821	0.611	0.206
Placebo	2.37 × 10^−8^ (1.51 × 10^−8^)	-	2.19 × 10^−8^ (1.05 × 10^−8^)			

* Log-transformed values. Values are means (SD). NC: nutraceutical combination BruMeChol^TM^*; p*-value for one-way repeated measures ANOVA for continuous variables (group = treatment allocation; time = baseline, 6 weeks, and 12 weeks), *p* < 0.05, by ANOVA simple effect analyses with Bonferroni correction.

## Data Availability

The data that support the findings of this study are available from the corresponding author, [ARB], upon reasonable request.
